# Protein phosphatase 4 promotes Hedgehog signaling through dephosphorylation of Suppressor of fused

**DOI:** 10.1038/s41419-020-02843-w

**Published:** 2020-08-11

**Authors:** Hengqing Liao, Jing Cai, Chen Liu, Longyan Shen, Xiaohong Pu, Yixing Yao, Bo’ang Han, Tingting Yu, Steven Y. Cheng, Shen Yue

**Affiliations:** 1 Department of Medical Genetics, Nanjing Medical University, Nanjing, 211166 China; 2 Department of Pathology, Liuzhou People’s Hospital, Liuzhou, 545006 China; 3 Jiangsu Key Laboratory of Xenotransplantation, Nanjing Medical University, Nanjing, 211166 China; 4 Department of Pathology, The Second Affiliated Hospital of Nanjing Medical University, Nanjing, 210037 China; 5 Departments of Pathology, Nanjing Drum Tower Hospital, The Affiliated Hospital of Nanjing University Medical School, Nanjing, 210008 China; 6 Jiangsu Key Lab of Cancer Biomarkers, Prevention and Treatment, Collaborative Innovation Center for Cancer Personalized Medicine, Nanjing Medical University, Nanjing, 211166 China

**Keywords:** Morphogen signalling, Phosphorylation

## Abstract

Reversible phosphorylation of Suppressor of fused (Sufu) is essential for Sonic Hedgehog (Shh) signal transduction. Sufu is stabilized under dual phosphorylation of protein kinase A (PKA) and glycogen synthase kinase 3β (GSK3β). Its phosphorylation is reduced with the activation of Shh signaling. However, the phosphatase in this reversible phosphorylation has not been found. Taking advantage of a proteomic approach, we identified Protein phosphatase 4 regulatory subunit 2 (Ppp4r2), an interacting protein of Sufu. Shh signaling promotes the interaction of these two proteins in the nucleus, and Ppp4 also promotes dephosphorylation of Sufu, leading to its degradation and enhancing the Gli1 transcriptional activity. Finally, Ppp4-mediated dephosphorylation of Sufu promotes proliferation of medulloblastoma tumor cells, and expression of Ppp4 is positively correlated with up-regulation of Shh pathway target genes in the Shh-subtype medulloblastoma, underscoring the important role of this regulation in Shh signaling.

## Introduction

Sonic Hedgehog (Shh) is an essential morphogenic and mitogenic factor that plays a key role in embryonic development and postnatal physiological processes^1,2^, regulating cell proliferation, differentiation, and patterning. Aberrant activation of Shh signaling can lead to hyper-proliferation and the development of malignancies^3,4^. A comprehensive understanding of the regulatory mechanisms of Shh signaling is essential for us to understand development as well as disease process.

Shh is the major ligand of vertebrate Hedgehog signaling pathway. Shh signaling is initiated by the binding of Shh ligand to the 12-pass transmembrane receptor Patched-1 (Ptch1)^5,6^, which attenuates the inhibition of Ptch1 on Smoothened (Smo)^7,8^, a G protein-coupled receptor, by promoting endocytic turnover of Ptch1 in lysosomes^9,10^. Smo moves into primary cilium and turn on the downstream transcription program orchestrated by three transcription factors Glioma-associated oncogene homolog 1, 2, and 3 (Gli1, Gli2, and Gli3)^11,12^. Gli1 is generally considered as an activator and Gli3 mostly as a repressor. Gli proteins share a highly conserved zinc-finger DNA-binding domain to bind a common *cis*-acting DNA element in target gene promoters^11,13^. In the absence of Shh signal, Gli1 is not transcribed, and Gli2/3 proteins are proteolytically processed into C-terminally truncated repressors (GliR), shutting off target gene expression. Activation of Shh signaling abrogates Gli processing, allowing full-length activators (GliA) to turn on target gene expression in the nucleus. Gli1 activator is also transcribed as a Shh target gene, and then autoregulates itself in a feed-forward loop. The output of Shh signal is determined by the ratio of GliA and GliR as the Gli code^14^.

Suppressor of fused (Sufu) acts as an essential regulator at the downstream of Shh pathway^15^. Sufu binds to all three Gli proteins, controlling their production, transport, and function^16–18^. Sufu is generally regarded as a negative regulator^19–21^, but our recent data unexpectedly show that Sufu is highly expressed in certain Shh-receiving tissues and is required for the maximal activation of Shh signaling^22,23^. Sufu accompanies GliA translocating into and GliR out of the nucleus. Trafficking of Sufu to the primary cilium is potentiated by GliA, but not GliR. These findings indicate that Sufu is required for every aspect of Gli functions as a molecular chaperone. Thus, the precise regulation of Sufu protein is important for controlling Shh signaling activity. Shh promotes poly-ubiquitination of Sufu, which leads to its degradation in proteasomes^24^. E3 ligase Skp1-Cul1-F-box protein Fbxl17 targets Sufu for its degradation in the nucleus^25^. Phosphorylation of Sufu at Ser-346 and Ser-342 by PKA and glycogen synthase kinase 3β (GSK3β) stabilizes Sufu against Shh-induced degradation^26^. However, the dephosphorylation of Sufu has not been reported.

Using immunoprecipitation mass spectrometry, we identified Protein phosphatase 4 regulatory subunit 2 (Ppp4r2) interacting with Sufu. Ppp4r2 is one of the regulatory subunits of protein phosphatase 4 (Ppp4), a member of the PPP family of serine/threonine protein phosphatases^27^. Ppp4 is a complex containing a catalytic subunit Ppp4c and several regulatory subunits Ppp4r1, Ppp4r2, Ppp4r3α, and Ppp4r3β^28,29^. These regulatory subunits recognize the different substrates and mediate the binding of substrates to Ppp4c, which catalyzes dephosphorylation. In *Drosophila* Hedgehog signaling, Ppp4 promotes the dephosphorylation of Smo^30^. Knockdown of Ppp4 and deletion of PP4-interaction domain in Smo both elevate Smo phosphorylation and Hh signaling activity^30^. Here, the interaction between Ppp4r2 and Sufu provides an opportunity for us to study the role of Ppp4 in mammalian Shh signaling. We show Ppp4r2 contributes to the dephosphorylation and turnover of Sufu upon Shh signaling, and promotes the proliferation of SHH-subtype medulloblastoma (MB) cells through modulating Sufu repressor activity.

## Materials and methods

### Cell lines, small interfering RNAs (siRNAs), and antibodies

Immortalized wild-type (WT) and *Gli2*^*−/−*^*;Gli3*^*−/−*^ mouse embryo fibroblasts (MEFs) were generous gifts from the Wade Bushman laboratory. *Ppp4r2*^*−/−*^ MEFs were derived from WT-MEFs and established using clustered regularly interspersed short palindromic repeat-CRISPR-associated protein 9 (CRISPR-Cas9) system. Gli-null MEFs were described before^22^ and derived from *Gli2*^*−/−*^*;Gli3*^*−/−*^ MEFs. HEK293, NIH3T3, and DAOY cells were purchased from ATCC. siRNAs were purchased from GenePharma (Shanghai, China), and their sequences are shown in Supplementary Table 1. The primary antibodies were rabbit anti-Sufu (Protein-Tech; 1:1000 for Western analysis, 1:100 for Immunofluorescence (IF) and immunocytochemistry of culture cell (ICC)), mouse anti-β-actin (Santa Cruz Biotechnology; 1:1000 for Western analysis), rabbit anti-Gli1 (Cell Signaling Technology; 1:1000 for Western analysis), goat anti-Gli3 (R&D; 1:500 for Western analysis), mouse anti-Flag (Sigma; 1:1000 for Western analysis, 1:500 for proximity ligation assay (PLA), and 1:200 for IF), mouse anti-Myc (Santa Cruz Biotechnology; 1:500 for Western analysis), rabbit anti-Ppp4r2 (Protein-Tech; 1:1000 for Western analysis and 1:100 for PLA), rabbit anti-Sufu-S342P (generated by Signalway Antibody, China; 1:1000 for Western analysis), mouse anti-Smo (Santa Cruz Biotechnology; 1:50 for IF), rabbit anti-Smo (ABclonal; 1:200 for Western analysis); mouse anti-acetylated α-tubulin (Sigma; 1:1000 for IF), and rabbit anti-Arl13b (Protein-Tech; 1:200 for IF). The secondary antibodies were horse radish peroxidase-conjugated antibodies from Santa Cruz Biotechnology (1:5000 for Western analysis) and Alexa Fluor-conjugated antibodies from Thermo Fisher Scientific (1:200 for IF).

### Mass spectrometry analysis of Sufu-binding proteins

NIH3T3 cells were cultured to confluence, and then were treated with Shh- or control-conditional medium for 24 h. Endogenous Sufu was immunoprecipitated with anti-Sufu antibodies. To validate our approach, the immunoprecipitates were analyzed by silver staining, or by Western blotting with anti-Gli3 and anti-Sufu. The eluate from immunoprecipitation was sent to gel-free mass spectrometry (Analysis Center, Nanjing Medical University).

### Immunoprecipitation

Cultured cells were lysed in radioimmunoprecipitation assay (RIPA) buffer (50 mM Tris-HCl, pH 7.4, 150 mM NaCl, 1 mM EDTA, pH 8.0, 1% NP-40, 0.5% sodium deoxycholate, and 1× Roche Complete protease inhibitor cocktail). The lysate was clarified by centrifugation for 20 min at 14,000 × *g* at 4 °C. Total protein was quantified using a Thermo Pierce Bicinchoninic Acid (BCA) Kit (Thermo), and 2000 μg of total protein was used for each precipitation by incubating at 4 °C overnight with indicated antibodies. The next day, add the prepared magnetic beads at 40 μl for each sample, and then incubate it at 4 °C for 4 h. The beads were then washed three times with a washing buffer (50 mM Tris-HCl, pH 7.4, 150 mM NaCl, 1 mM EDTA, pH 8.0, 0.5% NP-40, 10% glycerol) and eluted with sodium dodecyl sulfate sample buffer. The immunoprecipitates were analyzed by Western blotting.

### Fluorescent microscopy and determining subcellular localization of Sufu

Sufu-GFP was transfected alone or with Flag-Ppp4r2 into MEFs. The transfected cells were fixed with 4% paraformaldehyde (PFA) for 10 min at room temperature, and standard procedures for immunostaining were followed. Fluorescence-labeled proteins were visualized with a Carl Zeiss LSM710 microscope. The percentage of cells with mostly nuclear (N > C), mostly cytoplasmic (N < C), or evenly distributed (*n* = C) Sufu-GFP was calculated by randomly counting over 50 cells in each of the three triplicated glass coverslips.

### Proximity ligation assay

MEFs were seeded on glass coverslips in 12-well plates, and when the cells grew to 70% confluence, they were transfected with Flag-Sufu, then stimulated with ShhN-CM for 24 h. The cells were fixed in 4% formaldehyde, permeabilized with 0.3% Triton X-100, and stained with primary antibodies as standard immunofluorescent staining. PLA was carried out using Duolink in situ detection reagent (Sigma) following the manufacturer’s instructions. Microscopic images were captured under a Carl Zeiss LSM710 microscope using a ×63, 1.4 numerical aperture (NA) oil objective.

### CRISPR-Cas9 genome editing

Genome editing was achieved using the CRISPR-Cas9 technique in MEFs. Briefly, a single-guide RNA (sgRNA) targeted the first exon of mouse Ppp4r2 was designed and cloned into pX330 vector (from Addgene). The sgRNA sequence is: 5′-AGGCTGCAGGAGGAGGCGCTGAA-3′. Cells were transfected with CRISPR/Cas9 plasmids. At 48 h after transfection, transfection-positive cells were selected using G418 (500 μg/ml) for 5 days, followed by another 4 days without selection for expansion. After 5 days of selection, survived MEFs were seeded at 30 cells per well in 96-well plates and screened by Western blotting for Ppp4r2 protein level and sequencing for Ppp4r2 DNA mutation.

### Protein turnover assay

To measure protein turnover of endogenous Sufu, normal and CRISPR-edited MEFs were treated with cycloheximide (CHX; 10 μM; Sigma) to block protein synthesis. At the end of each time point, the cells were lysed in RIPA buffer for Western analysis.

### In vitro dephosphorylation assay

Phosphorylated Sufu was purified by anti-Myc immunoprecipitation from HEK293 cells transfected with Sufu-Myc and PKAc. Flag-tagged Ppp4r2 and Ppp4c were expressed from a coupled in vitro transcription/translation system (Promega) and purified by anti-Flag immunoprecipitation. Purified Sufu-Myc, Flag-Ppp4c, and Flag-Ppp4r2 were mixed in phosphatase buffer and incubated at 37 °C for 30 min. Then, the mixtures were analyzed by Western blotting.

### Immunofluorescence

MEFs with different genotypes were seeded on glass coverslips in 12-well plates and starved in DMEM (Dulbecco’s modified Eagle’s medium) containing 0.5% fetal bovine serum for 24 h before ShhN-CM treatment. The cells were fixed with 4% PFA, and standard procedures for immunostaining were followed. Confocal images of the primary cilium were acquired. The percentage of cells with cilia and the cilia length was calculated by randomly counting over 50 cells in each of the three triplicated glass coverslips. Quantification of the fluorescence intensity of Sufu and Smo in primary cilia was carried out using Image-Pro as described previously^10^.

### Immunocytochemistry of culture cells

To determine nucleocytoplasmic distribution of Sufu proteins, indicated MEFs were seeded on glass coverslips and treated with ShhN-CM for 24 h when the cell density reached 70%. The cells were then fixed, and standard procedures for immunocytochemistry staining were followed as described previously^22^. The percentage of cells with different cellular distribution of Sufu was calculated based on over 40 cells in three non-overlapping random fields.

### Luciferase assay

Cells were seeded in 6-well plates and transfected with the 8xGliBS-Luc reporter alone with Renilla control and indicated plasmids. Transfected cells were reseeded in 24-well plates before the luciferase activities were measured using the Dual Luciferase Reporter Assay Kit (Vazyme) following the manufacturer’s instructions. The firefly luciferase activity was normalized against the Renilla luciferase activity to correct for transfection efficiency of different groups.

### Reverse transcription (RT) and real-time PCR

Total RNAs were isolated from cultured cells with RNAiso Plus reagent (TaKaRa) and reverse transcribed using HiScript II Q RT SuperMix (Vazyme). Quantitative real-time PCR (qPCR) was carried out using AceQ qPCR SYBR Green Master Mix (Vazyme). Each measurement was repeated three times, and each sample was analyzed in triplicate with hypoxanthine phosphoribosyltransferase as an internal control. The qPCR primers are listed in Supplementary Table 2.

### Cell Count Kit-8 (CCK-8), 5-ethynyl-2′-deoxyuridine (EdU) incorporation, and colony formation assays

CCK-8 assay (APExBIO) was used according to the manufacturer’s instructions. The transfected cells were seeded into 96-well plates at 4 × 10^3^/well. At the indicated time, 10 μl of CCK-8 was added to each well, which contained 90 μl of medium. After incubation for 3 h, the optical densities at 450 nm of each well were measured using a microplate reader (Sunrise). Each sample had four duplicate wells and was independently performed in triplicate.

In EdU incorporation assay, cells were seeded at 5 × 10^4^ cells per well in 24-well plates and cultured for 36 h. Cells were incubated with 10 μM EdU for 4 h before the end of the culture period. Following culture, cells were fixed with 4% PFA, and then detected with Click-iT EdU Imaging Kit (Life Technologies, CA) according to the manufacturer’s procedure (Life Technologies, CA). The plates were visualized under ×20 magnification using Leica DMI 3000B with Leica DFC490 Digital Camera. Quantification of the percentage of EdU-positive cells was performed using the ImageJ program.

For the colony formation assay, 500 cells were plated in a P60 plate and allowed to grow until visible colonies appeared. Colonies were stained with Giemsa and counted.

### Statistical analysis

Statistical analyses were performed in the GraphPad Prism 5.0 environment. Each measurement was repeated at least three times. Comparisons between indicated groups were performed using independent-samples *t* test. *P* values <0.05 were considered statistically significant. *P < 0.05, **P < 0.01, and ***P < 0.001.

## Results

### Ppp4r2 is identified as a novel Sufu-binding protein

**Fig. 1:**
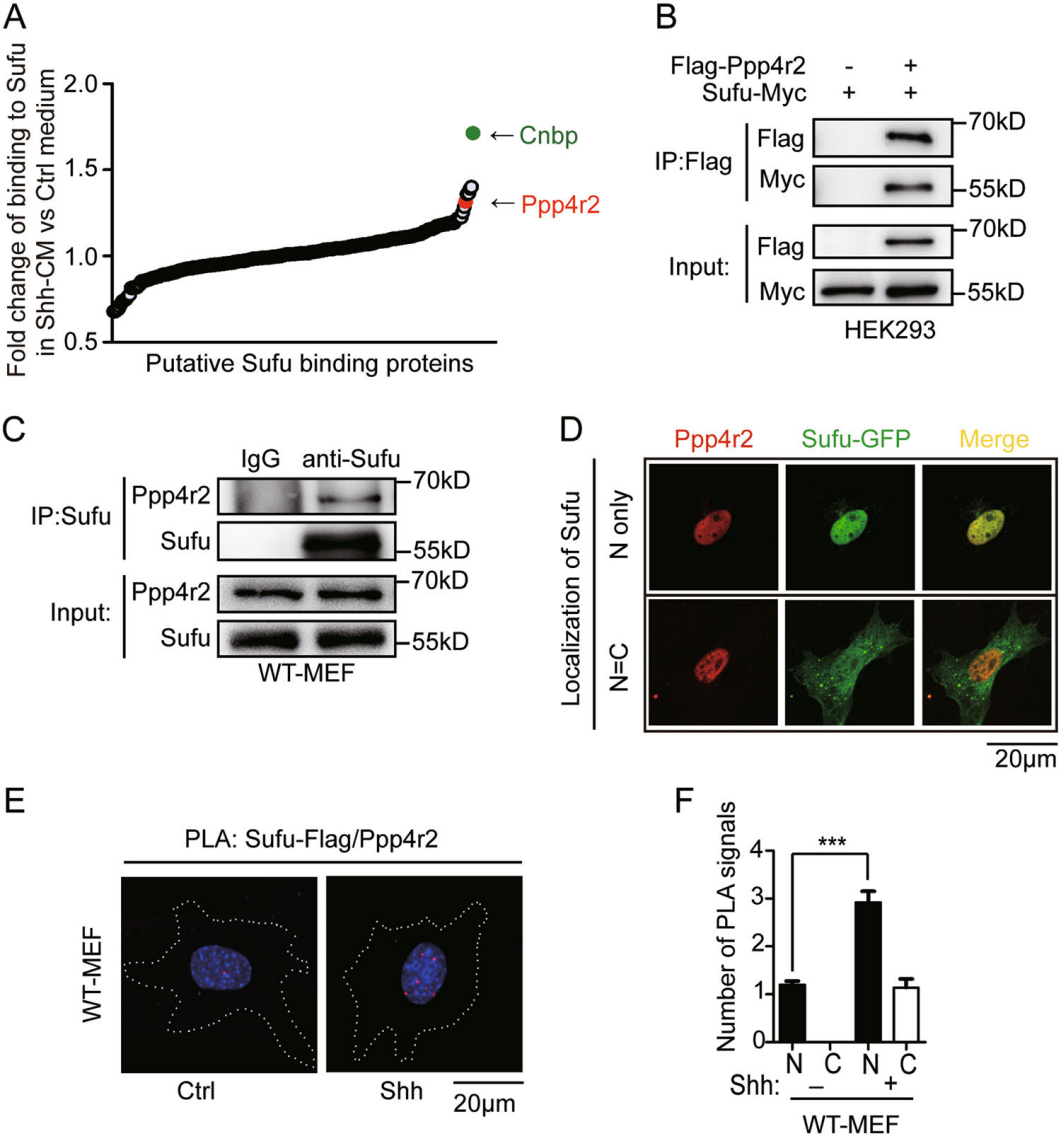
Ppp4r2 is identified as a Sufu-interacting protein. **a** Identified proteins in NIH3T3 cells interacted with Sufu in response to Shh by LC-MS/MS. **b** Co-immunoprecipitation experiment showing the interaction of Flag-Ppp4r2 and Sufu-Myc, which epitopically expressed in HEK293 cells. **c** Ppp4r2 was detected in Sufu immunoprecipitates from MEF cell lysates by Western blotting. **d** Immunofluorescence showed the co-localization of Sufu-GFP and Ppp4r2 in the nucleus. Sufu-GFP exhibited a mixed pattern of mostly nuclear localization (upper panel) or evenly nuclear-cytoplasmic distribution (lower panel). **e** PLA showing the numbers and localization of Sufu–Ppp4r2 complexes. MEFs were transfected with Flag-Sufu, and then treated with ShhN-CM for 24 h. The red PLA signal indicates the interaction of Sufu and Ppp4r2. The cell boarders were drawn with dotted lines. **f** Quantification of numbers of PLA signals in the nucleus and cytoplasm. At least 50 cells were counted at each data point. The error bars indicate SD. ***P < 0.001 (unpaired Student’s *t* test).

To understand the molecular mechanism by which Sufu is regulated in Shh signaling, we used liquid chromatography-tandem mass spectrometry to identify Sufu-interacting proteins in NIH3T3 cells, which are sensitive to the stimulation by Shh ligands. We treated NIH3T3 cells with Shh conditioned medium (Shh-CM) and performed immunoprecipitation using an antibody against the endogenous Sufu. Endogenous Gli3, a known Sufu-binding protein, was also captured in the immunoprecipitates, validating our approach (Supplementary Fig. S1A). Several binding protein candidates were isolated, including the regulatory subunit of serine/threonine protein phosphatase 4 (Ppp4r2) and zinc-finger protein CNBP (Fig. 1a). The interaction between CNBP and Sufu has been reported to be stabilized by Shh signaling in MEFs and MB^31^. Shh stimulation also stabilized the interaction between Ppp4r2 and Sufu (Fig. 1a), making it a strong candidate for a novel regulator of the Shh pathway.

To confirm the binding between Sufu and Ppp4r2, co-immunoprecipitation assay was done from lysates of HEK293 cells expressing both proteins. Sufu-Myc was detected in Flag-Ppp4r2 immunoprecipitates (Fig. 1b). To further confirm that their binding was not spurious interaction due to overexpression, we isolated endogenous Sufu from WT-MEFs. Endogenous Ppp4r2 was also detected in Sufu immunoprecipitates, confirming their interaction again (Fig. 1c). To map the specific region of Sufu for Ppp4r2 binding, Sufu deletion fragments were co-expressed with Ppp4r2. Both N- and C-termini of Sufu (1–267 amino acids (a.a.) and 331–483 a.a.) interact with Ppp4r2, but the middle region (250–350 a.a.) does not (Supplementary Fig. S1B). This result suggested that there may be some structural motifs present in both N- and C-termini of Sufu. We aligned the Sufu 1–267 and 331–483 fragments and found a common motif GPWL. However, C-terminal fragment of Sufu without GPWL still interact with Ppp4r2, indicating that GPWL is not necessary for Sufu to interact with Ppp4r2 (Supplementary Fig. S1C).

Immunofluorescence showed that endogenous Ppp4r2 mainly localized in the nucleus, and ectopically expressed Sufu-GFP distributed in both the cytoplasm and nucleus (Fig. 1d). Their nuclear localization provides an opportunity for interaction. To further certify their interaction in the nucleus, we performed the in situ PLA in MEFs ectopically expressed Flag-tagged Sufu and treated with Shh-CM. Using mouse anti-Flag, rabbit anti-Ppp4r2 antibodies and PLA probes, we detected specific PLA dots corresponding to the interaction between Sufu-Flag and Ppp4r2 (Fig. 1e). The PLA dots, which strictly localized in the nucleus, were enhanced by the Shh-CM treatment (Fig. 1f). Thus, our findings indicate that Shh contributes to Sufu interacting with Ppp4r2 in the nucleus. However, the role of Ppp4r2 binding with Sufu is still unknown.

### Ppp4r2 modulates the phosphorylation and turnover of Sufu

**Fig. 2:**
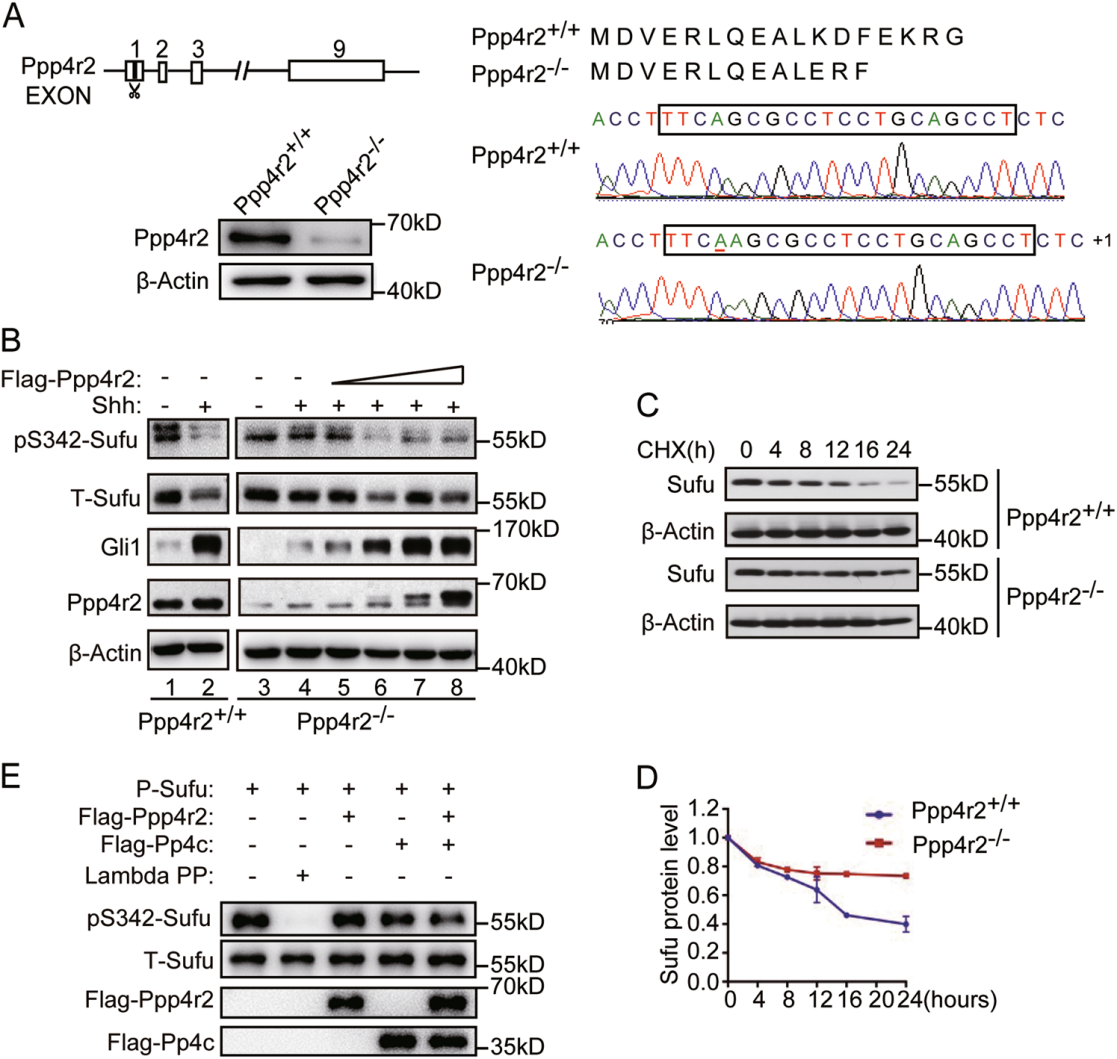
Knockout of Ppp4r2 stabilizes Sufu through regulating phosphorylation of S342 site. **a** *Ppp4r2* gene was CRISPR-edited in MEFs. Genomic structure of mouse *Ppp4r2* gene showing location of sgRNA in the first exon (left-upper). Sanger sequence of *Ppp4r2* gene in CRISPR-edited cells. An insertion of T at 29 of coding sequence resulted in frameshift mutation and the translation termination by as a stop codon soon after the inserted site (right). Western analysis evaluating Ppp4r2 protein levels in selected individual clones edited by CRISPR (left-lower). **b** Western analysis showing an increase in phosphorylation of Sufu-S342 site and a decrease in Shh-induced Gli1 expression in *Ppp4r2*^*−/–*^ cells. Re-introduction of Flag-Ppp4r2 decreased the phosphorylated and total Sufu in *Ppp4r2*^*−/−*^ MEFs, and restored Shh-induced Gli1 expression as well. Western analysis (**c**) and quantification thereof (**d**) showing stabilization of Sufu in *Ppp4r2*^*−/−*^ cells from two individual clones. Protein synthesis was blocked with cycloheximide (CHX) treatment. **e** Ppp4 dephosphorylates Sufu in vitro. Purified Sufu-Myc, Flag-Ppp4c, and Flag-Ppp4r2 were incubated in in vitro phosphatase buffer at 37 °C for 30 min. Sufu and P-Sufu were detected by Western blotting. Lambda PP lambda phosphatase, as a positive control.

To assess the function of Ppp4r2 on Sufu phosphorylation, CRISPR-Cas9 genome editing was used to knockout Ppp4r2 in MEFs. CRISPR-Cas9 sgRNAs targeting the first exon of Ppp4r2, which is located on mouse chromosome 6, were generated (Fig. 2a), and then introduced into MEF cells. A frameshift mutation was found in the coding sequence of Ppp4r2 in edited cells by Sanger sequencing (Fig. 2a). Western blotting confirmed the loss of Ppp4r2 protein expression in edited MEFs (Fig. 2a). Those cells were hereinafter referred as *Ppp4r2*^*−/−*^ MEFs.

We previously demonstrated that Shh signaling could normally bypass Sufu inhibition by promoting its degradation through the ubiquitin-proteasome pathway^24^. PKA and GSK3β sequentially phosphorylate Sufu at Ser-346 (S346) and Ser-342 (S342) sites^26^. Phosphorylation of Sufu at S346 and S342 could stabilize Sufu against Shh-induced degradation^26^. Given the crucial role of phosphorylation for Sufu stability, we tested its phosphorylation at S342 site and protein stability in *Ppp4r2*^−^^*/*−^ MEFs. Indeed, our results showed a reduction of Sufu protein level in WT-MEFs after Shh-CM treatment. Shh led to a decrease in the phosphorylation of Sufu at S342 site, making Sufu unstable (Fig. 2b, lanes 1 and 2). However, the decrease of phosphorylated and total Sufu could not be induced by Shh activation in *Ppp4r2*^−*/*−^ MEFs (Fig. 2b, lanes 3 and 4). In order to exclude the possible off-target effect of CRISPR-Cas9 editing, we re-introduced Flag-Ppp4r2 into *Ppp4r2*^−*/*−^ MEFs with a gradient dose (0.125–1 μg) to rescue the protein activity. The re-introduction rescued Shh-induced dephosphorylation and turnover of Sufu (Fig. 2b, lanes 5–8), demonstrating the involvement of Ppp4r2 in Shh-induced dephosphorylation of Sufu. Blocking protein synthesis with CHX showed that the turnover rate of Sufu was slower in *Ppp4r2*^*−/−*^ MEFs than in *Ppp4r2*^*+/+*^ MEFs (Fig. 2c, d). These data demonstrated that Ppp4r2 promoted the dephosphorylation and following degradation of Sufu in Shh signaling pathway.

To further determine whether Ppp4 can dephosphorylate Sufu directly, an in vitro phosphatase assay was performed using in vitro expressed Ppp4 subunits Ppp4c and Ppp4r2 and immunopurified phosphorylated Sufu proteins. Phosphorylated Sufu was purified by anti-Myc immunoprecipitation from HEK293 cells transfected with Sufu-Myc and PKAc. Purified Sufu proteins were incubated with Flag-Ppp4c and/or Flag-Ppp4r2 in a phosphatase buffer and followed by Western blotting with anti-p-S342-Sufu antibody. It was shown that phosphorylation levels of Sufu induced by PKAc was reduced when Sufu was co-incubated with Ppp4c and Ppp4r2 (Fig. 2e), indicating that Ppp4 directly dephosphorylates Sufu.

### Ppp4r2 promotes Shh signaling through dephosphorylating Sufu

**Fig. 3:**
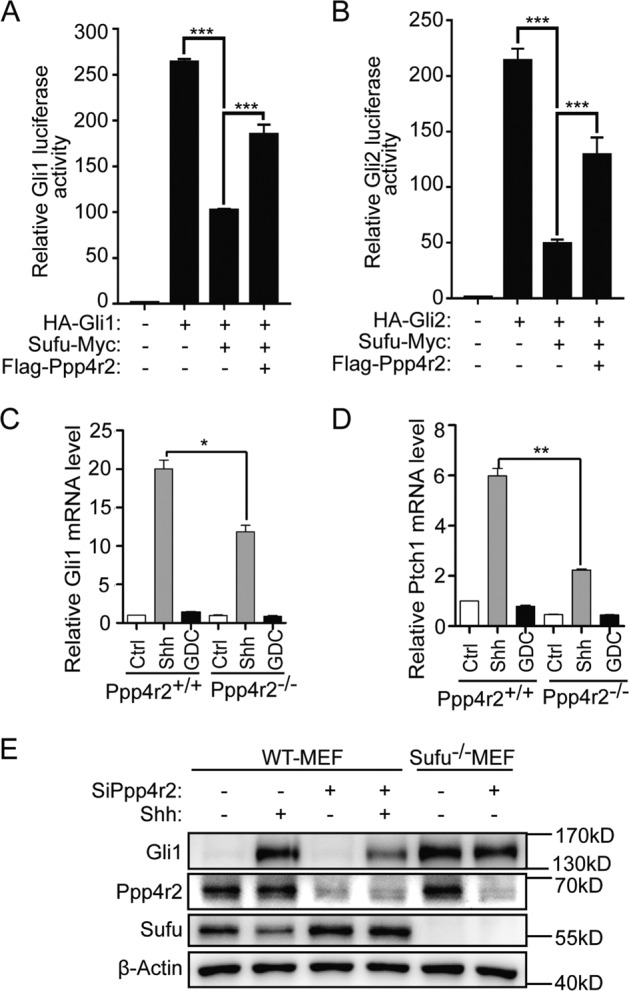
Knockout of Ppp4r2 inhibits Hh response in MEFs. Evaluation of Ppp4r2 activity on Gli1- (**a**) or Gli2- (**b**) mediated Hh response using luciferase reporter assay. *8×GliBS-luc* reporter served as the readouts, *Renilla* luciferase as an internal control. Each data point represents results from triplicate wells. Q-PCR detection of Hh target genes *Gli1* (**c**) and *Ptch1* (**d**) mRNA levels in clonal Ppp4r2 cells and unedited control cells. MEFs were treated with ShhN-CM and GDC-0449 for 24 h as indicated. **e** Western analysis of Gli1 expression in WT and *Sufu*^*−/−*^ MEFs transfected with Ppp4r2 siRNA. WT-MEFs were treated with Shh-CM to induce Gli1 expression. The error bars indicate SD. *P < 0.05; **P < 0.01; ***P < 0.001 (unpaired Student’s *t* test).

Since Ppp4r2 dephosphorylates Sufu in the Shh pathway, we need to understand the changes in Shh signal output of *Ppp4r2*^*−/−*^ MEFs. We firstly evaluated its effect on Gli activity by an *8×GliBS-luc* reporter assay. Introduction of Gli1 or Gli2 in NIH3T3 resulted in *8×GliBS-luc* reporter activation. Co-expression of Sufu severely inhibited Gli-mediated Hh reporter activation. However, its inhibitory effect on Gli activators was suppressed by Ppp4r2 (Fig. 3a, b). Then, we quantified the transcription responses to Shh ligand stimulation in *Ppp4r2*^*−/−*^ MEFs by quantitative RT-PCR (RT-qPCR), and found that Shh induction of target genes *Gli1* and *Ptch1* were apparently inhibited in *Ppp4r2*^*−/−*^ MEFs compared with *Ppp4r2*^*+/+*^ MEFs (Fig. 3c, d). Western blotting also showed marked curtailment of Gli1 activation in *Ppp4r2*^*−/−*^ background (Fig. 2b). More importantly, re-introduction of Ppp4r2 into knockout MEFs rescued Shh induction of Gli1 expression dose-dependently (Fig. 2b), demonstrating the ability of Ppp4r2 to positively modulate Shh signaling. Knockdown of Ppp4r2 with siRNA also inhibited Gli1 activation in WT-MEFs shown by Western blotting (Fig. 3e). However, this inhibition did not occur in *Sufu*^*−/−*^ MEFs (Fig. 3e), indicating that Ppp4r2 positively modulates Shh signaling through Sufu.

S342 site of Sufu is continuously phosphorylated in *Ppp4r2*^*−/−*^ MEFs regardless of Shh stimulation. To evaluate the effect of S342 phosphorylation on Sufu repressor activity, we did *8×GliBS-luc* reporter assay again. The Sufu-S342/6D mutant, mimicking phosphorylation, showed more repressor activity than WT Sufu. While the non-phosphorylatable Sufu-S342/6A mutant showed less repressor activity (Supplementary Fig. S2B). Even if we used Crm1-mediated nuclear export inhibitor LMB to gather Sufu mutants in the nucleus (Supplementary Fig. S2A), Sufu-S342/6D mutant still showed more repressor activity (Supplementary Fig. S2B), indicating that the phosphorylation status of Sufu determines its repressor activity, independent of its cellular distribution.

### Cellular distribution of Sufu mainly depends on Gli, but not on its phosphorylation

**Fig. 4:**
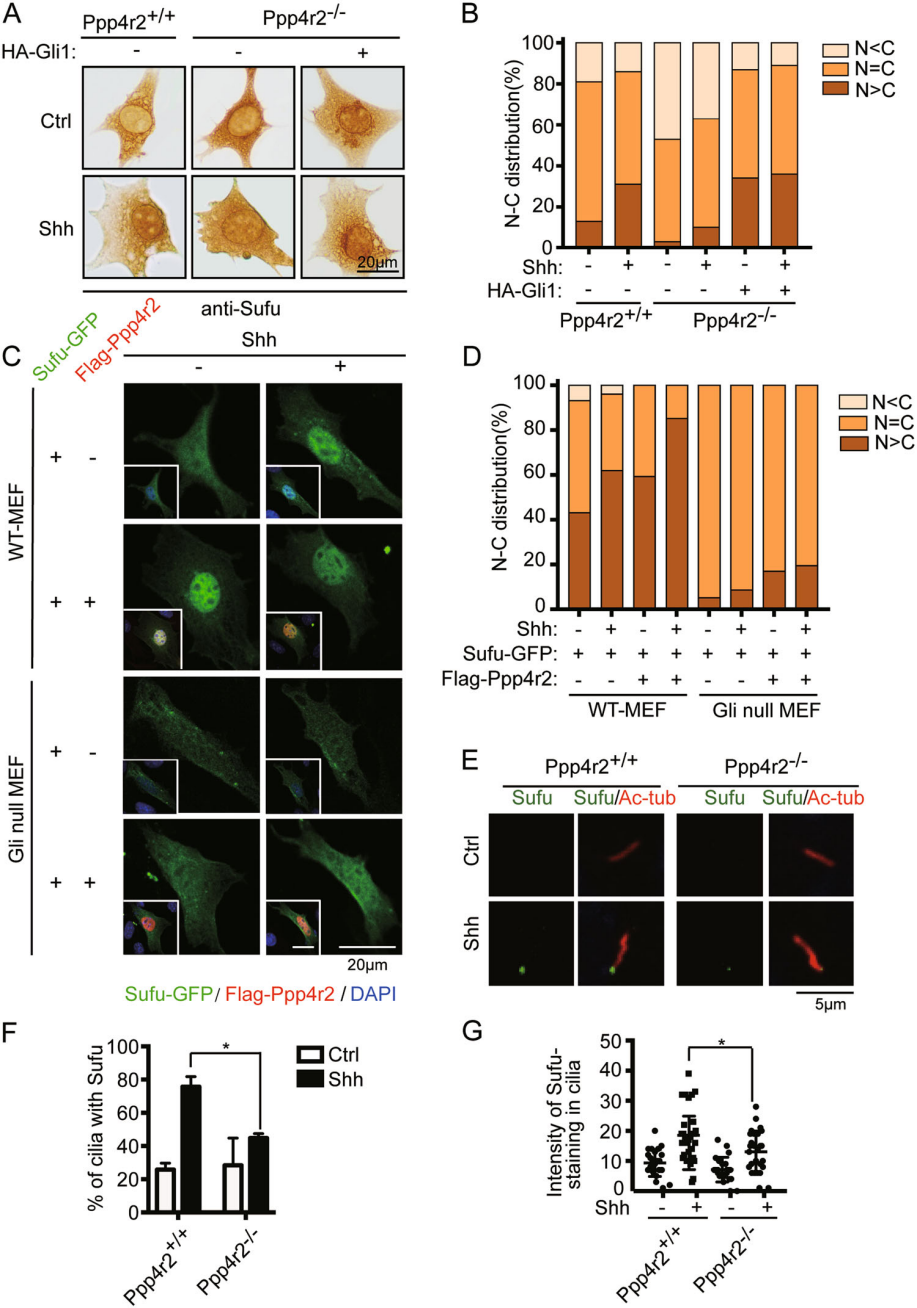
Sufu fails to accumulate at the nucleus and cilium tips in Ppp4r2 knockout cells under Shh stimulation. **a** Representative ICC images of Sufu in *Ppp4r2*^*+/+*^ and *Ppp4r2*^*−/−*^ MEFs. HA-Gli1 was transfected in indicated groups. Cells were treated with control or ShhN-CM for 24 h. **b** Quantification of Sufu subcellular distribution presented in **a**. Representative immunofluorescence images (**c**) and quantification of nuclear-cytoplasmic distribution (**d**) of Sufu-GFP in WT and Gli-null MEFs. Sufu-GFP was transfected into MEFs alone or together with Flag-Ppp4r2. At least 50 cells were counted at each data point in ICC and IF assay. **e** Representative immunofluorescence images of Shh-induced localization of endogenous Sufu at ciliary tips in *Ppp4r2*^*+/+*^ and *Ppp4r2*^*−/−*^ MEFs. Quantification showing percentage of Sufu-positive cilia (**f**) and the intensity of Sufu in primary cilia (**g**) decreased in *Ppp4r2*^*−/−*^ MEFs. *P < 0.05 (unpaired Student’s *t* test).

Sufu has a nuclear export signal in adjacent to the dual PKA-GSK3 phosphorylation site. We previously reported that phospho-mimicking Sufu-S342/6D mutant failed to bind Crm1 and accumulated in the nucleus of normal MEFs^22^. Here, we devised an experiment to determine the influence of Ppp4r2 on Sufu movement using *Ppp4r2*^*−/−*^ MEFs. Immunostaining showed that Sufu exhibited a mixed pattern of mostly nuclear (N > C), mostly cytoplasmic (N < C), or evenly distributed (*n* = C) in *Ppp4r2*^*+/+*^ MEFs (Fig. 4a, b) as we reported before^22^. Shh-CM treatment induced the nuclear localization of Sufu, but changed the percentage of cells with nuclear localization of Sufu from 13.1 to 31.3% (Fig. 4b). Interestingly, few untreated *Ppp4r2*^*−/−*^ cells showed abundance of Sufu in the nucleus. Shh-CM treatment hardly induced the enrichment of Sufu in the nucleus of *Ppp4r2*^*−/−*^ MEFs, but changed the percentage of cells with nuclear localization of Sufu only from ~0.6 to 1.6% (Fig. 4a, b). Conversely, co-expression of Ppp4r2 enriched Sufu-GFP in the nucleus, and Shh-CM treatment further increased the enrichment (Fig. 4c, d). Furthermore, we noticed that Shh hardly induced Gli1 expression in *Ppp4r2*^*−/−*^ MEFs (shown in Fig. 2b). Sufu mainly accompanies Gli activators translocating into the nucleus and Gli repressors out of the nucleus as a Sufu–Gli complex. Therefore, we speculated that the cytoplasmic retention of Sufu in *Ppp4r2*^*−/−*^ MEFs may be due to the loss of Gli1.

To prove our hypothesis, we introduced HA-Gli1 into *Ppp4r2*^*−/−*^ MEFs and observed cellular localization of Sufu again. Introduction of Gli1 dramatically induced abundance of Sufu in the nucleus of *Ppp4r2*^*−/−*^ MEFs, regardless of whether it was treated with or without Shh-CM (Fig. 4a, b). Furthermore, we found that Sufu-GFP was evenly distributed in Gli-null cells, which did not express all three Gli transcription factors as described before^22^. Neither Ppp4r2 introduction nor Shh treatment could manipulate the cellular distribution of Sufu-GFP in it (Fig. 4c, d). Although Sufu-S342/6D-GFP is more localized in the nucleus, while Sufu-S342/6A-GFP is more localized in the cytoplasm when transiently expressed in WT-MEFs (Supplementary Fig. S3B), they evenly distributed in Gli-null cells (Supplementary Fig. S3B). These data demonstrated that the cellular distribution of Sufu mainly depends on Gli transcription factors it accompanies not on its phosphorylation modification.

We and other groups previously demonstrated that Shh induces Sufu trafficking to the tip of the primary cilium^26,32^, which is potentiated by Gli activators and coupled to its nuclear import^22^. This trafficking is also failed in *Ppp4r2*^*−/−*^ MEFs. We examined ciliary Sufu staining in *Ppp4r2*^*−/−*^ MEFs and found that knockout of Ppp4r2 abolished the localization of Sufu at cilium tips (Fig. 4e). The percentage of cilia that was stained positive for Sufu and the intensity of Sufu staining in cilia was reduced in *Ppp4r2*^*−/−*^ MEFs compared to *Ppp4r2*^*+/+*^ MEFs (Fig. 4f, g). It is reported that Ppp4 is localized to the centrioles^33^, which is the basal portion of the primary cilium, so it is possible that ciliary defects disturb Sufu turnover in *Ppp4r2*^*−/−*^ MEFs. To exclude this possibility, we measured the physical properties of primary cilium in *Ppp4r2*^*−/−*^ MEFs after 30 h of low serum treatment to induce cilia growth (Supplementary Fig. S4A). Nearly 80% of cells formed a cilium based on our counting under microscope (Supplementary Fig. S4B). The length of the cilia is comparable to those formed in WT-MEFs (Supplementary Fig. S4C). Although the ciliary trafficking of Sufu was affected, we found that the ciliary transport of Smo was not changed in the absence of Ppp4r2 (Supplementary Fig. S4D, F). These data suggested that the failures of Shh-induced Sufu translocation in *Ppp4r2*^*−/−*^ MEFs are due to the loss of Gli activators, but not dysfunction of upstream factors.

### Ppp4r2 negatively regulates the tumor suppressor activity of Sufu

**Fig. 5:**
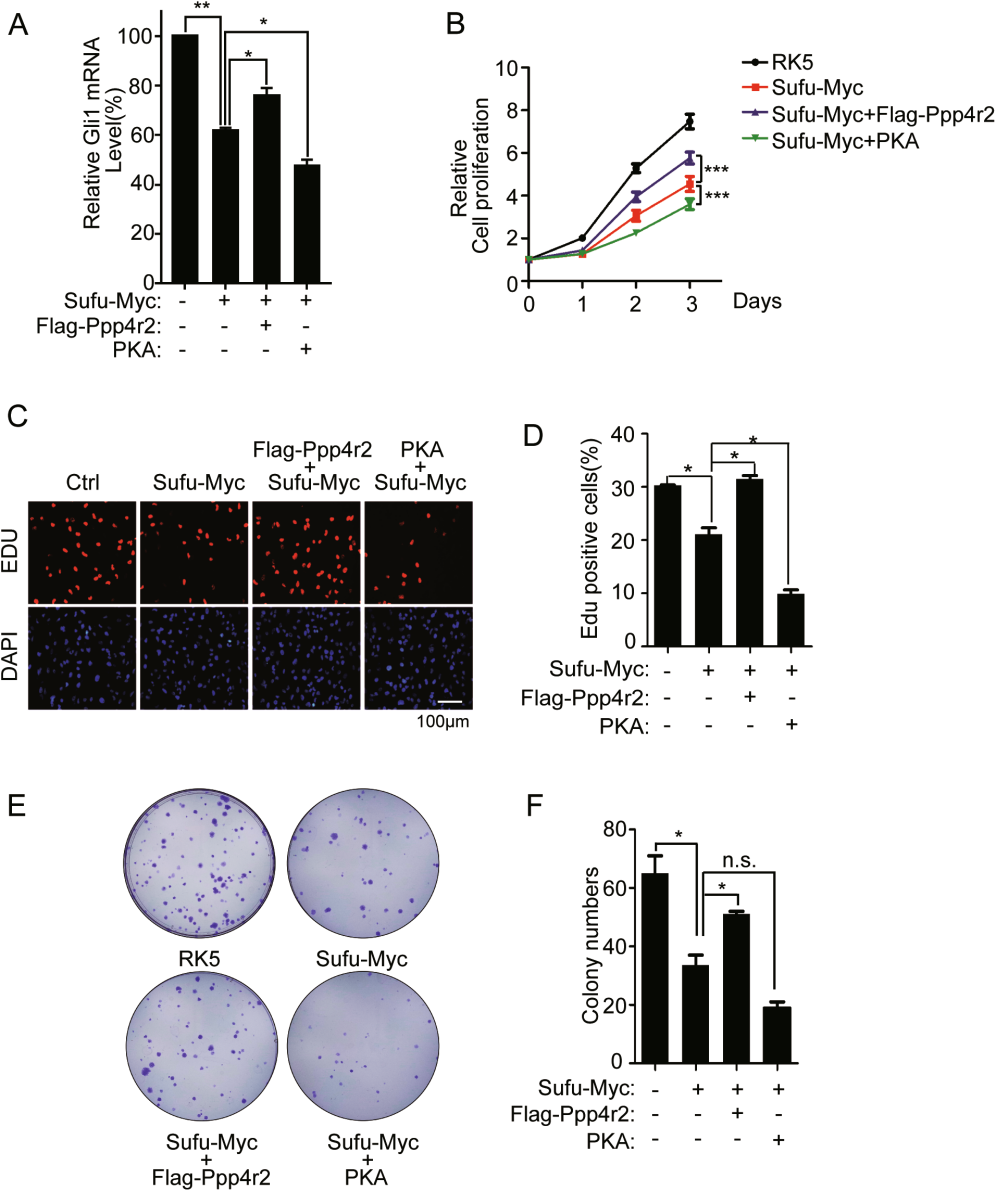
Ppp4r2 attenuates tumor repressor activity of Sufu in DAOY cell growth. **a** Q-PCR detection of Hh target gene *Gli1* mRNA levels in DAOY cells expressing Sufu with or without Ppp4r2 and PKA. **b** CCK-8 assays for DAOY cells in 96-well plates. **c** Fluorescence images and **d** percentage quantification of EdU incorporation assays as in **b**. The cells were assayed in 24-well plates. **e** Five hundred cells were cultured in P60 dishes and allowed to grow until visible colonies appeared. The plates were then fixed and stained with crystal violet for colony counting. **f** Quantification of **e**. *P < 0.05; **P < 0.01; ***P < 0.001; n.s. not significant (unpaired Student’s *t* test).

Sufu is a tumor suppressor gene mutated in MB^21^, which is the most common malignant pediatric brain tumor derived from cerebellar granule cell precursors. According to the transcriptional profiles, four different subtypes of MB have been described, that is, WNT, SHH, group C, and group D^34,35^. Aberrant activation of Shh signal is a leading cause of SHH-subtype MB. DAOY is a representative cell line of SHH-subtype MB, whose Gli1 messenger RNA (mRNA) levels continued to increase with the prolongation of Shh treatment (Supplementary Fig. S5A). Introduction of Sufu inhibited the expression of Gli1 mRNA in DAOY (Fig. 5a). This inhibition was promoted by co-introduction of PKAc and suppressed by Ppp4r2 (Fig. 5a). Given the relevant role of Ppp4r2 on Gli1 in Shh signaling, we further investigated the effect of Ppp4r2 in DAOY cell growth. DAOY cells expressing Myc-tagged Sufu grew slower as evident by CCK-8 assay (Fig. 5b). Co-expression of Ppp4r2 with Sufu in this assay repressed Sufu-mediated grow inhibition, while co-expression of PKAc reinforced the Sufu effect (Fig. 5b). Consistent with cell counting result, Ppp4r2 functioned as a cellular antagonist of the Sufu tumor suppressor, while PKA did as an agonist in DAOY cells, according to EdU incorporation (Fig. 5c, d) and colony formation results (Fig. 5e, f).

**Fig. 6:**
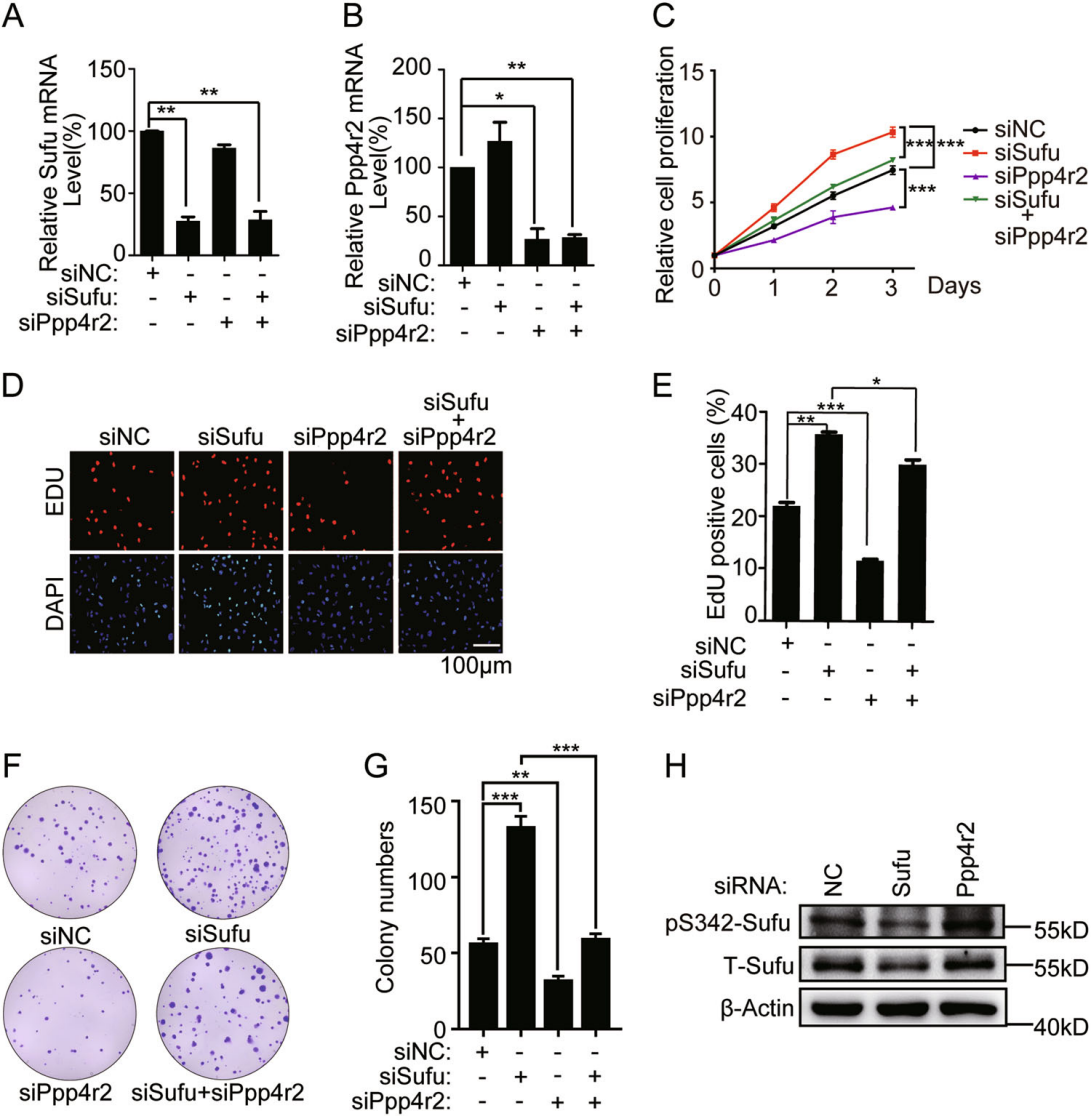
Knockdown of Ppp4r2 inhibits DAOY cell growth by accumulating Sufu. Q-PCR quantification of Sufu (**a**) and Ppp4r2 (**b**) mRNA levels in DAOY cells transfected with siRNA. **c** CCK-8 assays for DAOY transfected with indicated siRNA in 96-well plates. **d** Fluorescence images and **e** percentage quantification of EdU incorporation assays as in **c**. **f** Colony counting of DAOY cells expressing siRNA cultured in P60 dishes. **g** Quantification of **f**. **h** Western analysis showing the levels of phosphorylated and total Sufu in DAOY cells expressing siRNA. *P < 0.05; **P < 0.01; ***P < 0.001 (unpaired Student’s *t* test).

Next, we evaluated whether siRNA depletion of Ppp4r2 impairs DAOY cell growth. The knockdown efficiency of siSufu and siPpp4r2 was validated by RT-qPCR (Fig. 6a, b). The proliferation of DAOY cells was hampered by Ppp4r2 siRNA according to CCK-8 and EdU incorporation assay (Fig. 6c–e). DAOY cell knockdown of Ppp4r2 also exhibited reduced capacity to form colonies, as both the number and the size of foci were markedly reduced compared to those exhibited by control cells (Fig. 6f, g). Ppp4r2 siRNA increased the phosphorylation level of Sufu in DAOY (Fig. 6h). Importantly, the cell growth defect induced by Ppp4r2 reduction was dependent on Sufu, as double knockdown of Ppp4r2 and Sufu could restore the proliferation in DAOY (Fig. 6c–g).

In the process of dephosphorylation, Ppp4r2 recognizes the substrate, while Ppp4c removes phosphates from the substrate^27^. Interestingly, the proliferation of DAOY cells was not hampered, but promoted by Ppp4c siRNA (Supplementary Fig. S5D–F). DAOY cell knockdown of Pp4c also exhibited enhanced capacity to form colonies (Supplementary Fig. S5G, H), although Ppp4c siRNA increased the phosphorylation level of Sufu as Ppp4r2 siRNA did in DAOY (Supplementary Fig. S5I). Interestingly, the depletion of Ppp4c, but not Ppp4r2, induced the accumulation of Smo protein (Supplementary Fig. S5I), while the transcript level of Smo did not change (Supplementary Fig. S5J). These data suggested that Ppp4c silencing promotes DAOY proliferation mainly due to the accumulation of Smo. It has been reported that PP4 dephosphorylates Smo in *Drosophila* Hh signaling^30^, we speculated that the siPpp4c accumulated Smo by suppressing Smo dephosphorylation to increase its stability.

Given the relevant effect of Ppp4r2 in Hh pathway and MB, we searched the Gene Expression Omnibus (GEO) from NCBI database, and analyzed the mRNA expression of PPP4R2 and other Shh components in a published MB expression profiling study (285 primary MB samples; GEO accession: GSE37382)^36^. mRNA levels of *PPP4R2* and *PPP4C* were found to be increased in the SHH MB (Supplementary Fig. S6A, B). In addition, as shown in Table 1, Spearman’s correlation analysis indicated that the expression level of *PPP4R2* was positively correlated with *GLI1* (*r* = 0.379, *P* = 0.000) and *PTCH1* (*r* = 0.275, *P* < 0.001). The expression level of *PPP4C* was also positively correlated with *GLI1* (*r* = 0.557, *P* = 0.000) and *PTCH1* (*r* = 0.472, *P* = 0.000). Meanwhile, expression levels of *PPP4R2/PPP4C* both positively correlated with *SFRP1*, a molecular marker of SHH MB, and with *DKK1*, a marker of WNT MB. However, they were not significantly correlated with *NPR3*, a group C marker, or with *KCNA1*, a group D marker (Table 1). These results suggest that PPP4 complex promotes the transcriptional activity of Shh signaling and development of SHH-subtype MB.

**Table 1:** GSE37382 dataset (*n* = 285) correlations between *PPP4R2/PPP4C*, *GLI1/PTCH1*, and medulloblastoma biomarkers.

	*GLI1*	*PTCH1*	*DKK1*	*SFRP1*	*NPR3*	*KCNA1*	PPP4R2	*PPP4C*
*GLI1*								
Correlation coefficient		0.642	0.451	0.616	−0.108	0.011	0.379	0.557
*P* value		0.000	0.000	0.000	0.185	0.000	0.000	0.000
*PTCH1*								
Correlation coefficient	0.642		0.468	0.620	−0.180	0.089	0.275	0.472
*P* value	0.000		0.000	0.000	0.001	0.383	0.000	0.000
*DKK1*								
Correlation coefficient	0.451	0.468		0.466	0.052	−0.093	0.317	0.416
*P* value	0.000	0.000		0.000	0.758	0.000	0.000	0.000
*SFRP1*								
Correlation coefficient	0.616	0.620	0.466		−0.119	0.114	0.387	0.504
*P* value	0.000	0.000	0.000		0.004	0.265	0.000	0.000
*NPR3*								
Correlation coefficient	−0.108	−0.180	0.052	−0.119		−0.689	0.062	−0.009
*P* value	0.185	0.001	0.758	0.004		0.000	0.781	0.001
*KCNA1*								
Correlation coefficient	0.011	0.089	−0.093	0.114	−0.689		−0.130	−0.011
*P* value	0.000	0.383	0.000	0.265	0.000		0.006	0.380
*PPP4R2*								
Correlation coefficient	0.379	0.275	0.317	0.387	0.062	−0.130		0.375
*P* value	0.000	0.000	0.000	0.000	0.781	0.006		0.000
*PPP4C*								
Correlation coefficient	0.557	0.472	0.416	0.504	−0.009	−0.011	0.375	
*P* value	0.000	0.000	0.000	0.000	0.001	0.380	0.000	

## Discussion

Sufu plays critical roles in the production, trafficking, and function of Gli proteins in Hedgehog signaling. Previous studies illustrated that Sufu promotes the production of Gli repressors and restrains the activities of Gli activators. Recent literatures demonstrated that Sufu stabilizes Gli1 and accompanies its nuclear translocation for the maximal pathway activation. For now, we defined Sufu as a chaperone of all Gli proteins required for every aspect of Gli function. The balance of the controversial effects of Sufu in Hh signaling pathway may depend on the involvement of unknown Sufu-binding proteins or on different modifications of Sufu. Using affinity proteomics, we found a novel Sufu-interacting protein Ppp4r2 dephosphorylating Sufu. In particular, Shh signal promoted the interaction of Sufu with Ppp4r2 in nucleus. The interaction promoted the dephosphorylation and degradation of Sufu and downstream Gli1 transcriptional activity.

Protein phosphorylation is one of the most important post-translational modifications. Reversible phosphorylation is capable of affecting many aspects of the substrates, such as conformation, stability, activity, and protein interaction. Our previous study demonstrated that PKA and GSK3β sequentially phosphorylated S346 and S342 sites of mammalian Sufu and stabilized Sufu against Shh-induced degradation, thereby inhibiting Gli transcription activity. In this study, dephosphorylation at the dual sites by Ppp4 showed exactly converse effects. It was found that parathyroid hormone-like hormone promoted Sufu phosphorylation and stabilization in chondrocytes in the growth plate, thereby inhibiting Shh pathway activity and hypertrophic differentiation of chondrocytes^37^. These results indicate that reversible phosphorylation of Sufu is physiologically significant. In addition, NIMA-related expressed kinase 2A also stabilizes Sufu by promoting phosphorylation at T225 and S352 sites, consequently impairing the nucleocytoplasmic distribution and transcriptional activity of Gli2^38^.

In addition to Sufu, several core components of Hedgehog pathway undergo reversible phosphorylation mediated by protein kinases and phosphatases, which acts as an effective regulatory mechanism to modulate Hedgehog signal activities^39^. It has been well established that multiple sites’ phosphorylation on Ci/Gli by PKA, PKA-primed CK1, or PKA-primed GSK3 facilitates the production of truncated transcriptional repressor CiR/GliR through proteolytic processing^40–43^. When Hh signal is switched on, full-length Ci/Gli activators are subject to complete degradation catalyzed by E3 ligase HIB/SPOP^44,45^. The association between Ci/Gli and HIB/SPOP can be disrupted by CK1-mediated phosphorylation of Ci/Gli, as a consequence, protecting CiA/GliA from premature degradation^46^. In contrast, PP2A promotes Ci dephosphorylation and attenuates Ci cleavage, therefore, positively regulating Hh signaling outputs in *Drosophila*^30^. However, this mechanism has not been found in mammals^47^.

*Drosophila* Smo undergoes multiple phosphorylation at its carboxyl-terminal intracellular tail upon Hh stimulation^48^. PKA and CK1 sequentially phosphorylate three clusters of Ser/Thr residues in the intracellular tail of Smo, which stabilizes Smo on the cell surface and induces a conformational switch of Smo cytoplasmic tail from a closed inactive to an open active form^49,50^. PP1 and PP2A negatively modulate Smo phosphorylation and activity^51^. Ppp4 was also found to regulate the phosphorylation of Smo, which needs Cos2 to serve as a scaffold to associate Ppp4 and Smo^30^. Although the involved kinases and phosphatases may not be identical, the reversible phosphorylation of Smo in mammalian Hedgehog signaling pathway should be conserved. In this study, Ppp4 was also found to dephosphorylate Sufu upon Shh stimulation in mammalian cells. It suggests that Hh signal may dispatch Ppp4 to combine with different substrates to control the activity of downstream signals. Therefore, targeting the regulatory subunit rather than the catalytic subunit of the phosphatase can control the phosphorylation state of the targeted substrate more precisely.

**Fig. 7:**
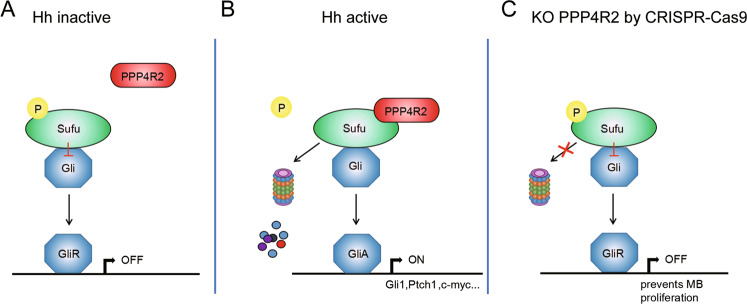
Ppp4r2 dephosphorylation of Sufu regulates Hh signaling. **a** Without Hh signal, PKA and GSK3β phosphorylate and stabilize Sufu, thereby repressing the pathway activation. **b** With Hh signal, Ppp4 dephosphorylates Sufu, thereby degrading Sufu and activating Gli activators. **c** Inhibition of Ppp4r2 prevents the degradation of Sufu.

In developmental signal pathway, protein phosphorylation has been revealed to play a critical role in precisely controlling the signal transduction^52^. Sufu is an essential regulator for both Gli activator and repressor, suggesting the appropriate modification of it is critical for normal transduction of Hh gradient. Based on our finding, we proposed a model to explain Sufu phosphorylation in Shh signaling. In this model, PKA and GSK3β phosphorylate and stabilize Sufu (Fig. 7a), and Hh signal promotes Ppp4 to dephosphorylate Sufu, thereby activating Gli activators (Fig. 7b). Deregulation of Sufu phosphorylation may result in malformations of Hh-dependent embryonic development, which remains to be further studied in animal models.

## Supplementary information

Supporting Information

Figure S1

Figure S2

Figure S3

Figure S4

Figure S5

Figure S6
