# Supplementary material for: Protein phosphatase 4 promotes Hedgehog signaling through dephosphorylation of Suppressor of fused
Source: Cell Death Dis. 2020 Aug 11;11(8):686. doi: 10.1038/s41419-020-02843-w (PMC7442787; doi:10.1038/s41419-020-02843-w)
Supplement: Supplementary file 1 — Supporting Information [file 41419_2020_2843_MOESM1_ESM.docx]

# Supporting Information

# Protein phosphatase 4 promotes Hedgehog signaling through dephosphorylation of Suppressor of fused

Hengqing Liao, Jing Cai, Chen Liu, Longyan Shen, Xiaohong Pu, Yixing Yao, Bo’ang Han, Tingting Yu, Steven Y Cheng and Shen Yue

Table S1: siRNAs sequences;

Table S2: The qPCR primers;

Figure legends of S1-S6.

Table S1. siRNA sequences

| Gene | Organisms | 5’-primer | 3’-primer |
| --- | --- | --- | --- |
| Ppp4r2 | Homo | GGUUGUUAGCUGUGUUUAUTT-F’ | AUAAACACAGCUAACAACCTT-R’ |
| Ppp4c | Homo | GCUACUGCACUGAGAUCUUTT-F’ | AAGAUCUCAGUGCAGUAGCTT-R’ |
| Sufu | Homo | CCUCAUUCCUCUCUGCCUATT-F’ | UAGGCAGAGAGGAAUGAGGTT-R’ |

Table S2. qPCR primers

| Gene | Organisms | 5’-primer | 3’-primer |
| --- | --- | --- | --- |
| Gli1 | *Mus* | CCCGGGTTATGGAGCAGCCAGA | CTGGCATCAGAAAGGGGCGAGA |
| Ptch1 | *Mus* | TGGCCGCATTGATCCCTATC | ACACAGGGGCTTGTGAAACA |
| Hprt | *Mus* | TATGGACAGGACTGAAAGAC | TAATCCAGCAGGTCAGCAAA |
| Ppp4r2 | *Homo* | TGTGATGGTTGTTAGCTGTGTT | GACCTCTCAGTATAGCTTGGTGA |
| Ppp4c | *Homo* | AAGGTTCGCTATCCTGATCGC | AGCCATAGACCTGCGTGATCT |
| Sufu | *Homo* | CCAATCAACCCTCAGCGGCAGAATG | GTAGGTGAGAAAGAGGGCTGTC |
| Actin | *Homo* | CATCGAGCACGGCATCGTCA | TAGCACAGCCTGGATAGCAAC |

**Figure S1. LC-MS/MS identified Ppp4r2 binding to Sufu.** (A) Western analysis showing endogenous Gli3 in anti-Sufu immunoprecipitates but not in control. (B) Western analysis showing Ppp4r2 binding to both the N- and C- terminal fragments of Sufu. Sufu 250-350 fragment failed to interact with Ppp4r2. (C) Western analysis showing Sufu 331-483 fragment deleted GPWL (Sufu-331-483-mut) still interact with Ppp4r2. Sufu 1-267 fragment and 331-483 fragment had a common motif GPWL.

**Figure S2. S342 phosphorylation effects on Sufu repressor activity independent of its cellular distribution.** (A) Representative immunofluorescence images of nuclear-cytoplasmic distribution of Sufu-GFP and its mutants in WT MEFs. (B) Evaluation of repressor activity of Sufu and its mutants on Gli1mediated Hh response using 8×*GliBS-luc* reporter assay. 8×*GliBS-luc* reporter served as the readouts, Rellina luciferase as an internal control. Each data point represents results from triplicate wells. LMB blocked nuclear export and accumulated Sufu in the nucleus.

**Figure S3. Gli is necessary for phosphorylated Sufu to accumulate in the nucleus.** (A) Representative immunofluorescence images of different cellular distribution of Sufu-GFP when transiently expressed in MEFs. Nucleus are stained with DAPI (blue) in insets. (B) Quantification of nuclear-cytoplasmic distribution of Sufu-GFP and its mutants in WT and Gli null MEFs.

**Figure S4. Ciliogenesis and ciliary localization of Smo was not influenced in Ppp4r2^-/-^ MEFs.** (A) Representative confocal images of anti-acetylated tubulin (red) showing primary cilium in Ppp4r2^-/-^ MEFs. Nucleus are stained with DAPI (blue). Quantitation of percentage (B) and ciliary length (C) of ciliated cells from (A). (D) Representative immunofluorescent staining of Smo (red) in primary cilium (green, stained by anti-Arl13b). Quantitation of percentage of Smo-positive primary cilia (E) and the intensity of Smo in primary cilia (F).

**Figure S5. Knockdown of Pp4c couldn’t inhibit DAOY cell growth. (A)** Q-PCR quantified Gli1 mRNA levels in DAOY cells treated by Shh-CM for different time durations. Q-PCR quantification of Pp4c (B) and Sufu (C) mRNA levels in DAOY cells transfected with siRNA. (D) CCK-8 assays for siRNA-transfected DAOY cells in 96-well plates. (E) Fluorescence images and (F) percentage quantification of EdU incorporation assays as in (D). (G) Colony counting of DAOY cells expressing siRNA cultured in P60 dishes. (H) Quantification of (G). (I) Western analysis showing the levels of Smo and Sufu in DAOY cells expressing siRNAs. (J) Q-PCR quantification of Ppp4r2, Pp4c, Smo and Sufu mRNA levels in DAOY cells expressing siRNAs.

**Figure S6. PPP4R2 and PP4C were up-regulated in SHH-subtype medulloblastoma.** GEO analysis of 285 medulloblastoma samples (GEO accession: GSE37382) showing mRNA expression of PPP4R2 (A) and PP4C (B) in different subtypes of medulloblastoma.
