# Supplementary figures and images for: Protein phosphatase 4 promotes Hedgehog signaling through dephosphorylation of Suppressor of fused
Source: Cell Death Dis. 2020 Aug 11;11(8):686. doi: 10.1038/s41419-020-02843-w (PMC7442787; doi:10.1038/s41419-020-02843-w)

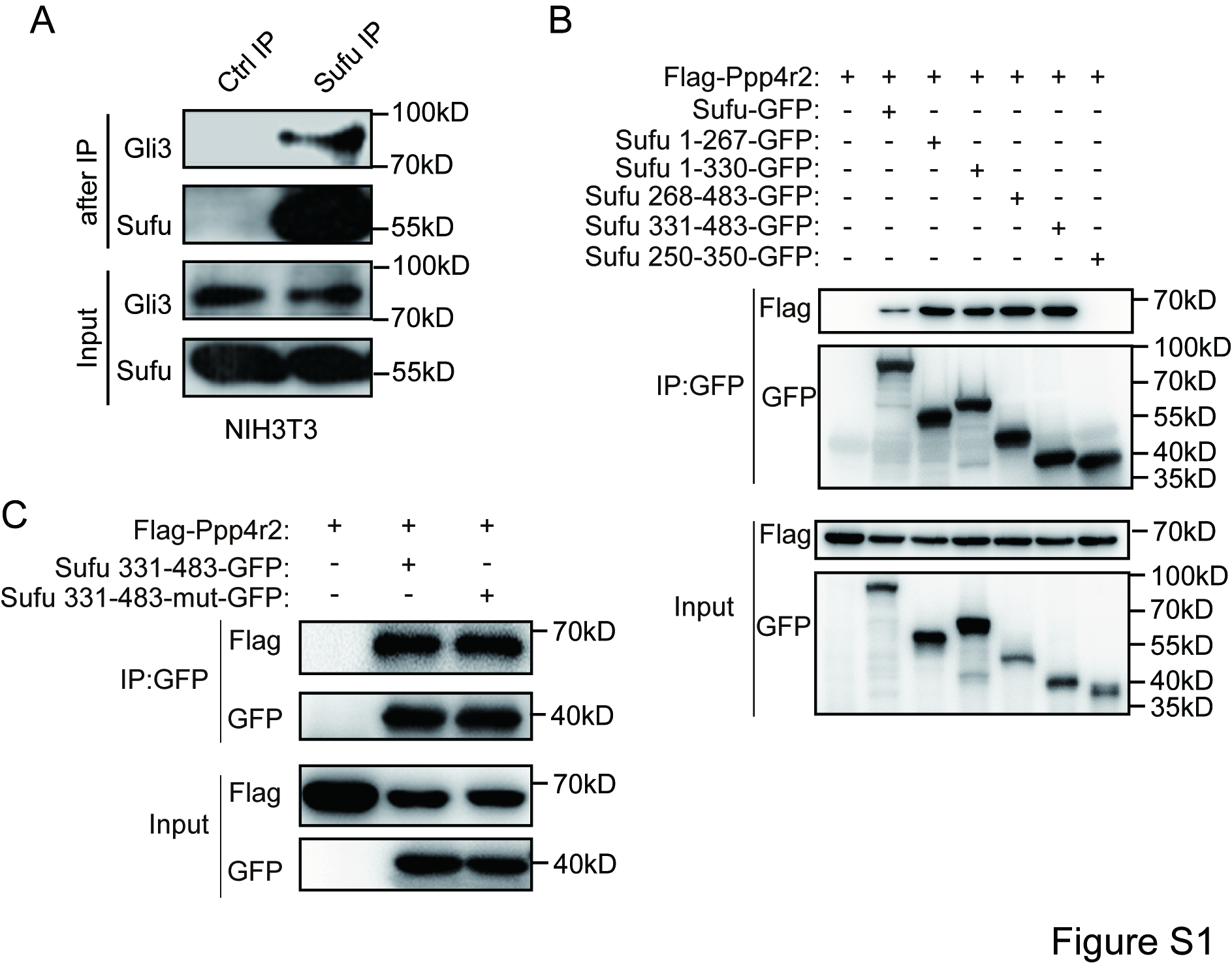

Supplement: Supplementary file 2 — Figure S1 [file 41419_2020_2843_MOESM2_ESM.tif]

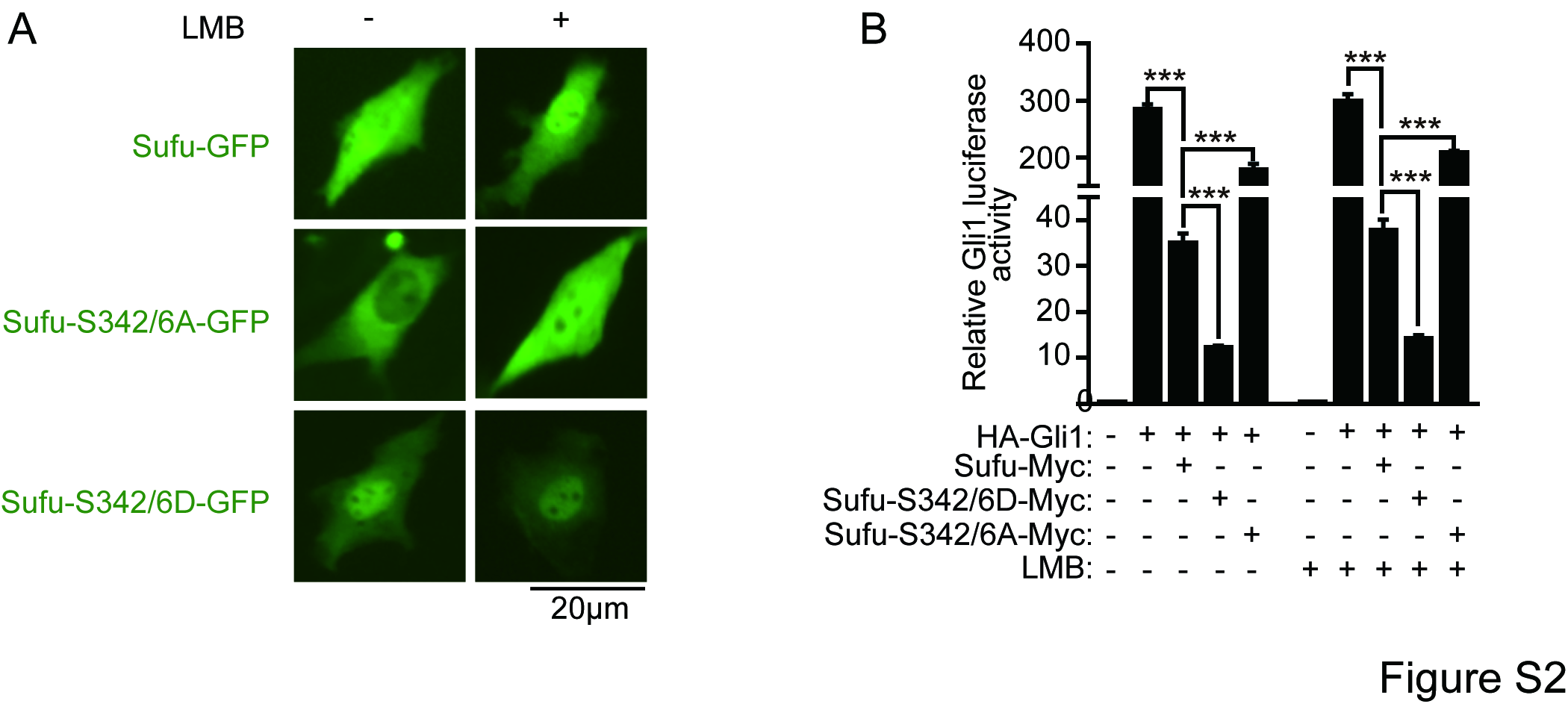

Supplement: Supplementary file 3 — Figure S2 [file 41419_2020_2843_MOESM3_ESM.tif]

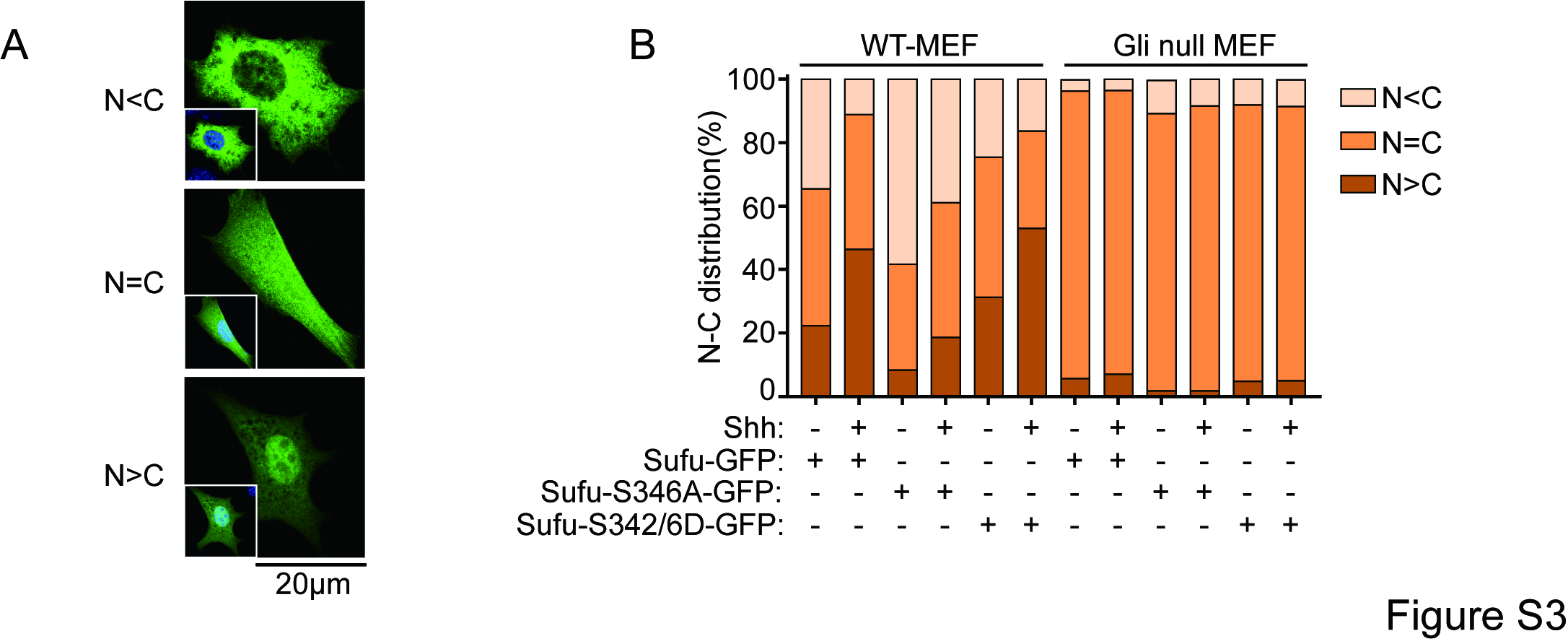

Supplement: Supplementary file 4 — Figure S3 [file 41419_2020_2843_MOESM4_ESM.tif]

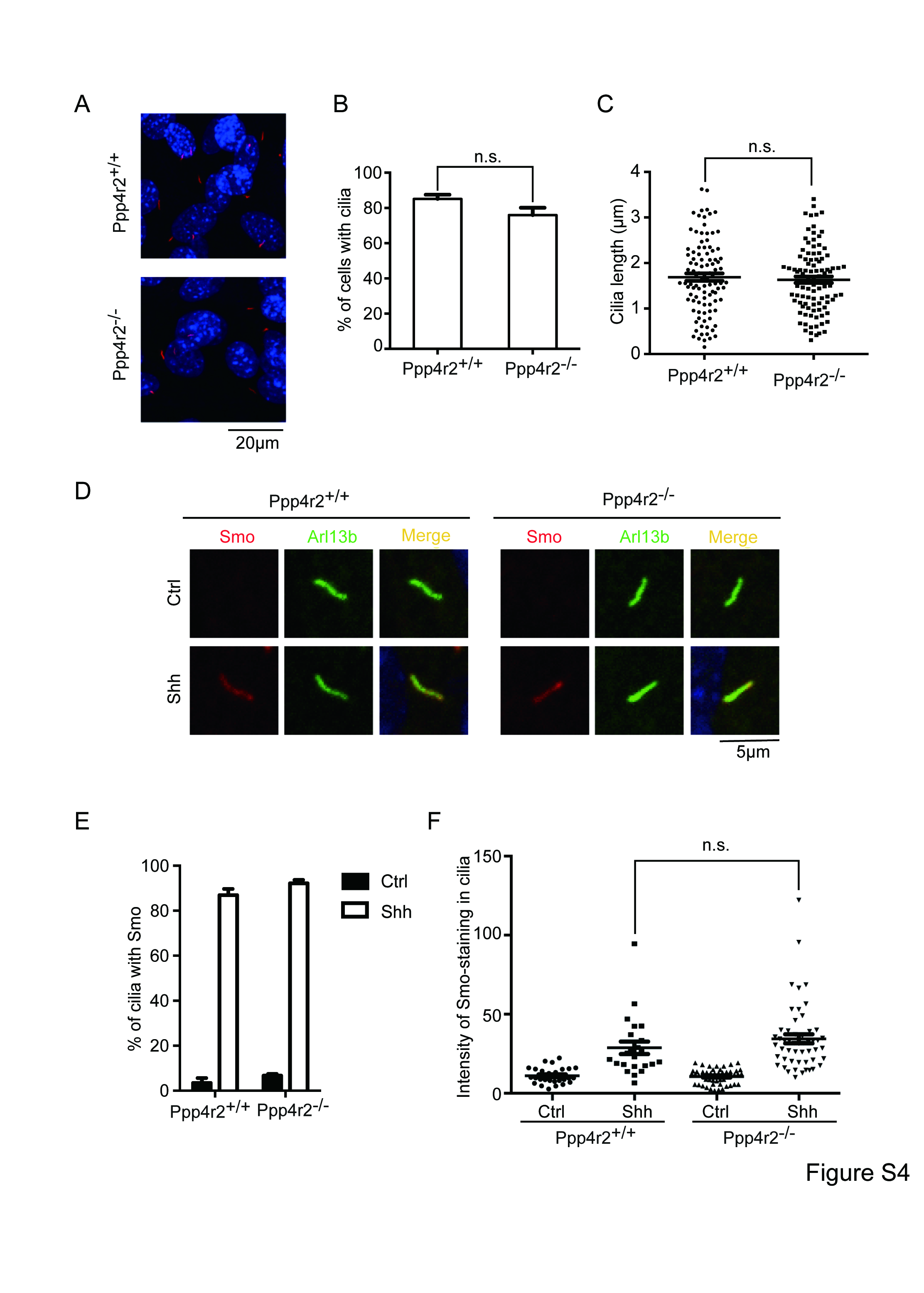

Supplement: Supplementary file 5 — Figure S4 [file 41419_2020_2843_MOESM5_ESM.tif]

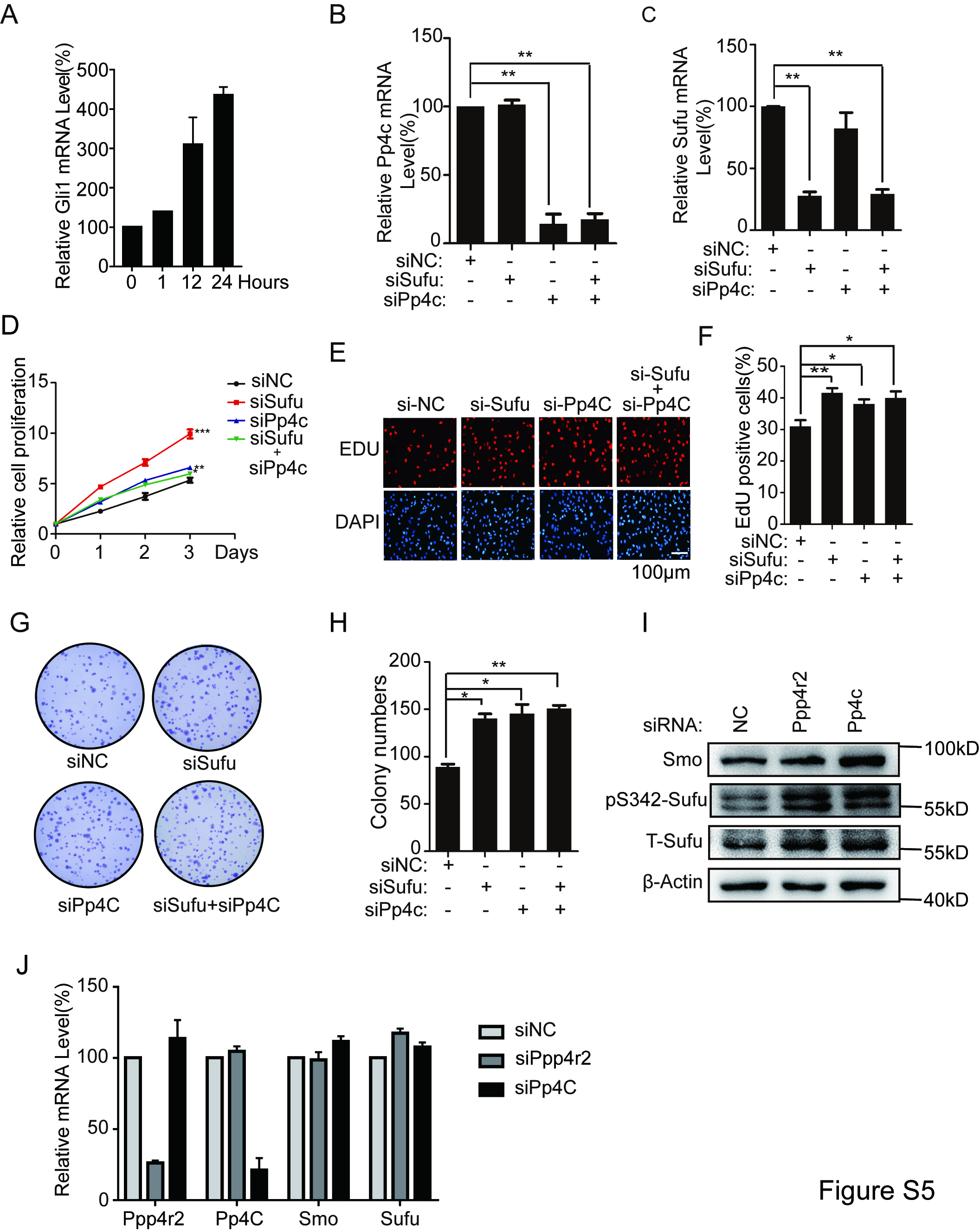

Supplement: Supplementary file 6 — Figure S5 [file 41419_2020_2843_MOESM6_ESM.tif]

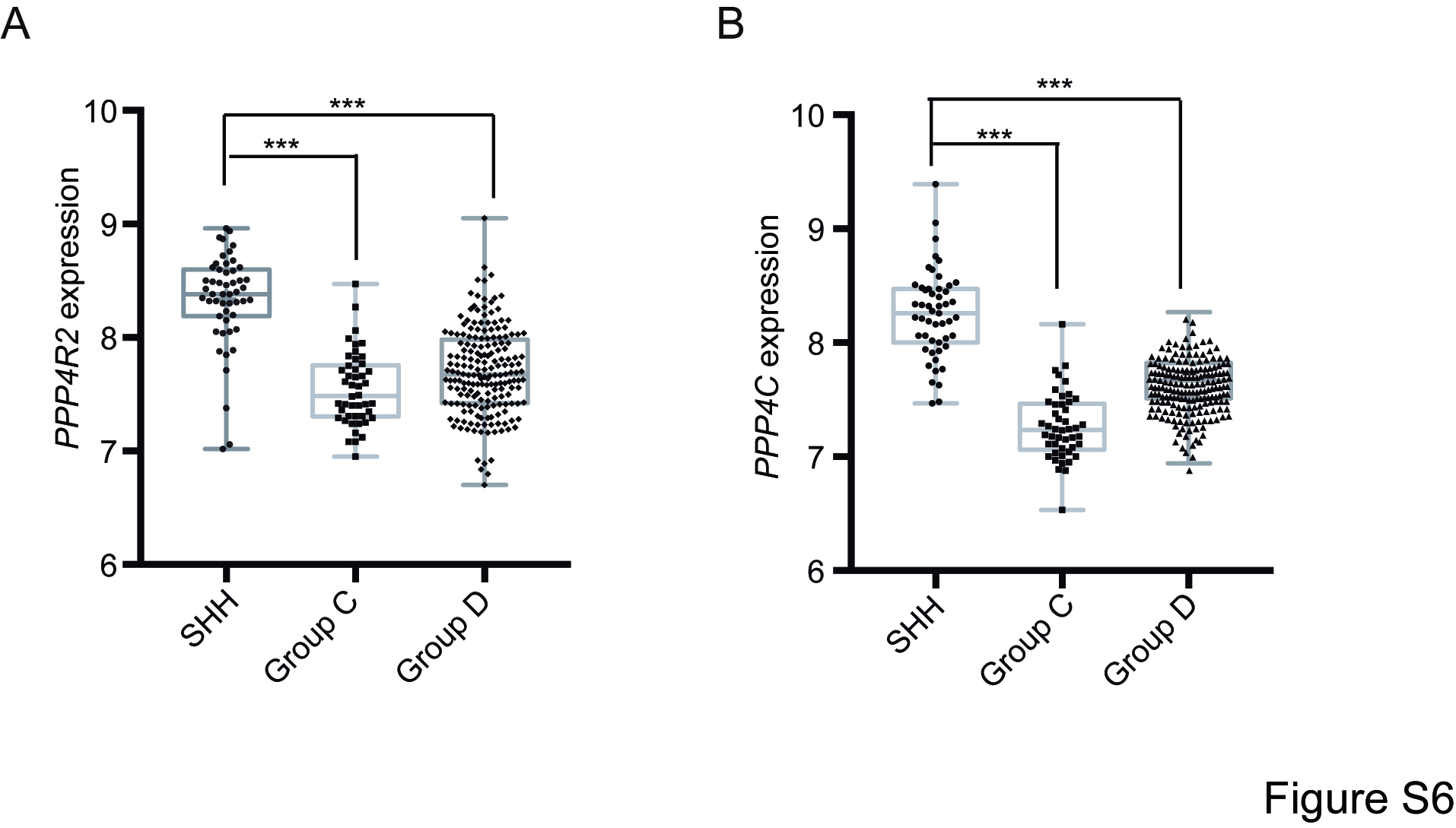

Supplement: Supplementary file 7 — Figure S6 [file 41419_2020_2843_MOESM7_ESM.tif]
